# Long-term outcomes of ablation, liver resection, and liver transplant as first-line treatment for solitary HCC of 3 cm or less using an intention-to-treat analysis: A retrospective cohort study

**DOI:** 10.1016/j.amsu.2022.103645

**Published:** 2022-04-20

**Authors:** T. Ivanics, L. Rajendran, P.A. Abreu, M.P.A.W. Claasen, C. Shwaartz, M.S. Patel, W.J. Choi, A. Doyle, H. Muaddi, I.D. McGilvray, M. Selzner, R. Beecroft, J. Kachura, M. Bhat, N. Selzner, A. Ghanekar, M. Cattral, B. Sayed, T. Reichman, L. Lilly, G. Sapisochin

**Affiliations:** a Multi-Organ Transplant Program, Division of General Surgery, Toronto General Hospital, University Health Network, University of Toronto, Toronto, ON, Canada; b Department of Surgery, Henry Ford Hospital, Detroit, MI, USA; c Department of Surgical Sciences, Akademiska Sjukhuset, Uppsala University, Uppsala, Sweden; d Division of General Surgery, Toronto General Hospital, University of Toronto, Toronto, ON, Canada; e Department of Surgery, Division of HPB & Transplant Surgery, Erasmus MC Transplant Institute, University Medical Centre Rotterdam, Rotterdam, the Netherlands; f Division of Surgical Transplantation, Department of Surgery, University of Texas Southwestern Medical Center, Dallas, TX, USA; g Joint Department of Medical Imaging, Mount Sinai Hospital and University Health Network, University of Toronto, Toronto, ON, Canada; h Department of Radiology, Toronto General Hospital, University of Toronto, ON, Canada; i Multi-Organ Transplant Program, Toronto General Hospital Research Institute, University Health Network, Toronto, ON, Canada; j Division of Gastroenterology, Department of Medicine, University of Toronto, Toronto, ON, Canada

**Keywords:** Transplantation, Resection, Ablation, Radiofrequency ablation, HCC, Intention to treat

## Abstract

**Background:**

Curative-intent therapies for hepatocellular carcinoma (HCC) include radiofrequency ablation (RFA), liver resection (LR), and liver transplantation (LT). Controversy exists in treatment selection for early-stage tumours. We sought to evaluate the oncologic outcomes of patients who received either RFA, LR, or LT as first-line treatment for solitary HCC ≤ 3 cm in an intention-to-treat analysis.

**Materials and methods:**

All patients with solitary HCC ≤ 3 cm who underwent RFA, LR, or were listed for LT between Feb-2000 and Nov-2018 were analyzed. Cox regression analysis was then performed to compare intention-to-treat (ITT) survival by initial treatment allocation and disease-free survival (DFS) by treatment received in patients eligible for all three treatments.

**Results:**

A total of 119 patients were identified (RFA n = 83; LR n = 25; LT n = 11). The overall intention-to-treat survival was similar between the three groups. The overall DFS was highest for the LT group. This was significantly higher than RFA (p = 0.02), but not statistically significantly different from LR (p = 0.14). After multivariable adjustment, ITT survival was similar in the LR and LT groups relative to RFA (LR HR:1.13, 95%CI 0.33–3.82; p = 0.80; LT HR:1.39, 95%CI 0.35–5.44; p = 0.60). On multivariable DFS analysis, only LT was better relative to RFA (LR HR:0.52, 95%CI 0.26–1.02; p = 0.06; LT HR:0.15, 95%CI 0.03–0.67; p = 0.01). Compared to LR, LT was associated with a numerically lower hazard on multivariable DFS analysis, though this did not reach statistical significance (HR 0.30, 95%CI 0.06–1.43; p = 0.13)

**Conclusion:**

For treatment-naïve patients with solitary HCC ≤ 3 cm who are eligible for RFA, LR, and LT, adjusted ITT survival is equivalent amongst the treatment modalities, however, DFS is better with LR and LT, compared with RFA. Differences in recurrence between treatment modalities and equipoise in ITT survival provides support for a future prospective trial in this setting.

## Highlights

• Ablation, resection, and transplantation can cure hepatocellular carcinoma (HCC).
• For patients with solitary HCC≤3 cm controversy exists in treatment selection.
• Ablation, resection, and transplantation have similar intention-to-treat survival.
• The disease-free survival is better with liver resection and liver transplantation.

## Abbreviations

AFP: Alpha-fetoprotein
BCLC: Barcelona Clinic Liver Cancer
ECOG PS: Eastern Cooperative Oncology Group Performance Status
CI: Confidence interval
DFS: Disease-free survival
ECOG: Eastern Cooperative Oncology Group
ETOH: Alcohol-related liver disease
HBV: Hepatitis B virus
HCC: Hepatocellular carcinoma
HCV: Hepatitis C virus
HR: Hazard ratio
ITT: Intention-to-treat
IQR: Interquartile range
LT: Liver transplantation
LR: Liver resection
MELD: Model for End-Stage Liver disease
OS: Overall survival
RFA: Radiofrequency ablation
UNOS: United Network for Organ Sharing
US: United States
SEER: Surveillance, Epidemiology, and End Results Program
STROBE: Strengthening the Reporting of Observational studies in Epidemiology
QALY: Quality Adjusted Life Years

## 1 Introduction

Hepatocellular carcinoma represents the leading cause of cancer-related deaths in many parts of the world and is estimated to become the third most common cause of cancer-related deaths by 2040 [1,2]. The incidence rate of HCC in countries with a high sociodemographic index, such as the United States, has increased since the 1990 [1].

Acceptable first-line treatment for early-stage hepatocellular carcinoma (BCLC-0 [single ≤ 2 cm] or BCLC-A [up to 3 nodules ≤ 3 cm], with preserved liver function and Eastern Cooperative Oncology Group [ECOG] performance status 0) include radiofrequency ablation (RFA), liver resection (LR), and liver transplantation (LT) [[3], [4], [5]]. Nonetheless, controversy exists in the curative-intent treatment selection for early-stage (≤3 cm) single tumours, as all three treatment modalities offer favourable results, each with moderate-high evidence [6,7]. Due to practical and ethical concerns, a randomized trial has not been performed for management of these patients. Furthermore, previous studies have compared at the most two of these available modalities [[8], [9], [10], [11], [12]].

We sought to evaluate the oncologic outcomes of patients who received either RFA, LR, or LT as first-line treatment for single HCC ≤ 3 cm in an intention-to-treat analysis. The rationale for the solitary HCC cohort was to limit heterogeneity in prognosis due to potentially varying tumour biology.

## 2 Material and methods

This study was approved by our institutional Research Ethics Board (REB #16-5285), and a waiver of informed consent was obtained. This study complies with the Strengthening the Reporting of Observational Studies in Epidemiology (STROBE) statement for observational studies [13]. Moreover, this work has been reported in line with the Strengthening the Reporting of Cohort Studies in Surgery (STROCSS) criteria [14].

### 2.1 Study design and population

**Fig. 1:**
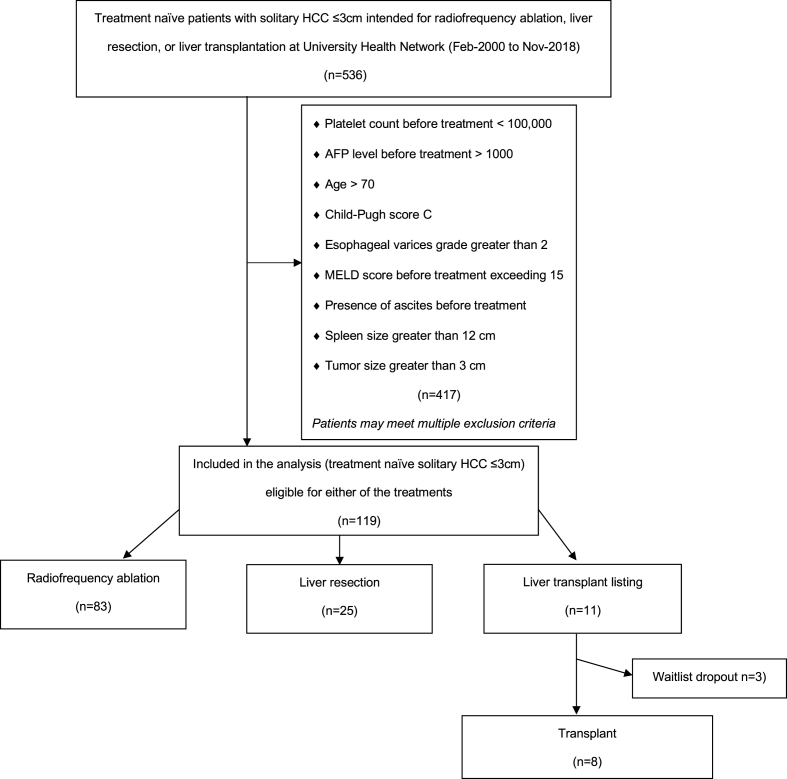
STROBE-compliant diagram of patient inclusion and exclusion.

This is a retrospective cohort study of patients from a single high-volume academic medical center. Adult (≥18 years) patients with solitary HCC ≤ 3 cm who underwent either RFA, LR, or were listed for an LT between Feb-2000, and Nov-2018 were included. The last day of follow-up was April 5, 2021. The diagnosis of HCC was made according to international guidelines [15]. The treatment selection was established by an institutional multidisciplinary board discussion and based on tumour size, location, liver function, patient comorbidities, and functional status. Further details of the treatment selection process are outlined elsewhere [6]. Patients were excluded if they had pathology other than HCC, had received previous treatments, or were not eligible for all of the three treatments (Fig. 1). In the study period, the treatment decision was based on consensus from a multidisciplinary discussion. Patients considered eligible for RFA were those with single HCC under 3 cm, acceptable liver function (Child-Pugh A or B), absence of encephalopathy, and a tumor being amenable to an imaging-guided procedure [6]. Furthermore, typically patients with advanced cirrhosis and portal hypertension were not considered for LR unless a laparoscopic approach and minor hepatic resection could be performed for treatment [16]. Moreover, with regards to LT, contraindications included an AFP level >1000, age greater than 70, and medical comorbidities that would preclude transplantation [6]. To ensure inclusion of patients theoretically eligible for either RFA, LR, or LT we excluded patients with a platelet count <100,000 before treatment [17], AFP level >1000 before treatment [18], age >70 years [6], Child-Pugh score C^19^, esophageal varices grade greater than 2 [19], Model for End-stage Liver Disease (MELD) score before treatment exceeding 15 [20], presence of ascites or encephalopathy pretreatment [21], and a spleen size exceeding 12 cm [17].

### 2.2 Covariates

We recorded gender (male or female); age; liver disease etiology; biologic MELD score; and pre-treatment platelet count (x1000), Child-Pugh score (A or B), and alpha-fetoprotein (AFP) (ng/dL). We defined pre-treatment as the most recent measurement (no longer than 6 months) before RFA, LR, or LT listing. Tumours were categorized as deep (≥2 cm or would require a resection greater than a wedge) or superficial (<2 cm or could be performed with a wedge resection) depending on the depth from the liver surface assessed on cross-sectional imaging (computed tomography or magnetic resonance imaging) on axial, sagittal and coronal sections.

### 2.3 Outcome measures

The study's primary outcome was intention-to-treat (ITT) overall survival and disease-free survival (DFS).

### 2.4 Intention-to-treat survival

ITT was evaluated from the first treatment modality that was selected for curative intent. In the case of.

RFA and LR this was recorded as the time of the treatment. In the case of LT, the intention-to-treat was recorded at the time of listing for transplantation. The ITT analysis thus accounted for patients who were placed on the waitlist but dropped out.

### 2.5 Follow-up, survival, and recurrence

After treatment, patients are followed with thoracoabdominal contrast-enhanced computed tomography (CT) and AFP measurements in 3-month intervals for the first 2 years, every 6 months in the 3rd and 4th year post-treatment, and yearly thereafter. Patients with cirrhosis will resume routine 6-month surveillance after 5 years post-treatment. If a recurrence is suspected, additional imaging studies are obtained, which include dedicated contrast-enhanced CT, contrast-enhanced ultrasonography, or magnetic resonance imaging (MRI) [22].

### 2.6 Statistical analysis

Descriptive data were expressed as medians and interquartile ranges (IQR) and compared using Mann-Whitney U tests. Categorical variables were expressed using numbers and percentages and compared using chi-square and Fischer exact tests. ITT survival was estimated using the Kaplan-Meier method stratified by RFA, LR, or LT listing. Patients were censored at death or last known follow-up. DFS was defined as the time after treatment during which the patient was alive and free of disease. For DFS, patients were censored at recurrence, death, or loss to follow up. DFS was estimated using the Kaplan-Meier method stratified by treatment using log-rank tests. For all survival analyses, pairwise comparisons using Benjamini-Hochberg correction were performed. Cox proportional hazards regression models were used for adjustment of a priori selected clinically relevant confounding variables including MELD score, tumor size, patient age, AFP level, year of treatment, and tumor location.

All two-sided p-values less than 0.05 were considered statistically significant. Statistical analyses were performed using R (version 4.1.1 2021, R Core Team R: A language and environment for statistical computing. R Foundation for Statistical Computing, Vienna, Austria. http://www.R-project.org/).

## 3 Results

### 3.1 Study population

A total of 119 patients met inclusion criteria (RFA n = 83, LR = 25, LT n = 11; Fig. 1). The dropout rate for patients listed for LT was 27% (n = 3). Reasons for dropout included death (n = 1), disease control with bridging therapy (n = 1), and patient request (n = 1). The median follow-up of the cohort was 6.6 years (IQR 3.1–10.5). There was no difference in the duration of median follow-up based on the treatment received: RFA 6.8 years (IQR 3.5–10.3), LR 4.5 years (IQR 2.2–7.4), and LT 8.4 years (IQR 3.7–12.9) (p = 0.17). Patients were similar in gender, age, etiology of liver disease, MELD score, Child-Pugh score, and AFP. Tumor size was highest in the LR group (Table 1). The clinical decision-making for the LT listed patients is shown in Table S1.

**Table 1:** Patient and tumor characteristics.

	RFA (N = 83)	LR (N = 25)	LT (N = 11)	p-value
**Male, n (%)**	66 (80%)	21 (84%)	8 (73%)	0.73
**Age, median (IQR)**	60 (56, 66)	64 (55, 67)	60 (52, 65)	0.78
**Etiology, n (%)**				
ETOH	6 (7%)	0 (0%)	1 (9%)	
HBV	46 (55%)	17 (68%)	5 (46%)	
HCV	22 (27%)	7 (28%)	4 (36%)	
NASH	4 (5%)	0 (0.0%)	0 (0%)	
Other	5 (6%)	0 (0%)	0 (0%)	
**MELD score, median (IQR)**	7 (6, 8)	7 (6, 8)	7 (7, 9)	0.15
**Tumor size (cm), median (IQR)**	2.0 (1.6, 2.5)	2.5 (2.3, 2.8)	1.7 (1.4, 2.1)	0.002
**Platelet count (x1000), median (IQR)**	154 (123, 192)	158 (137, 217)	128 (124, 145)	0.08
**Child-Pugh score, n (%)**				0.41
A	79 (95%)	25 (100%)	11 (100%)	
B	4 (5%)	0 (0%)	0 (0%)	
**AFP (ng/dL), median (IQR)**	6 (4, 42)	7 (4, 92)	9 (5, 146)	0.50
**Tumor location, n (%)**				0.99
Superficial	47 (57%)	14 (56%)	6 (55%)	
Deep	36 (43%)	11 (44%)	5 (46%)	

**Abbreviations:** AFP: alpha-fetoprotein, ETOH: alcohol-related liver disease, HBV: hepatitis B virus, HCV: hepatitis C virus, IQR: interquartile range, LR: liver resection, LT: liver transplant, MELD: Model for End-stage Liver Disease, NASH: non-alcoholic steatohepatitis, RFA: radiofrequency ablation.

### 3.2 Intention-to-treat survival

**Fig. 2:**
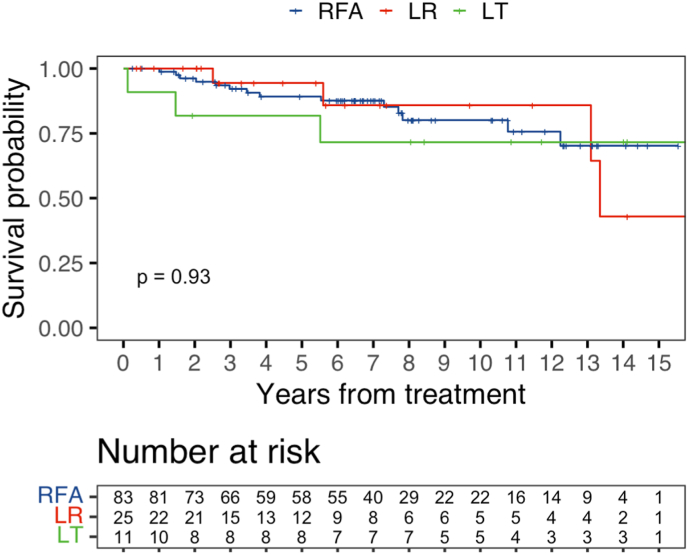
Intention-to-treat survival.

The median survival was not reached in the RFA group, was 13.3 years in the LR group, and was not reached in the LT group. The unadjusted ITT survival was similar among the groups: (%, 95% CI) 1-year RFA 100% (100-100), LR 100% (100-100), LT 90.9% (75.4–100) (p < 0.01), 5-year RFA 89.2% (82.4–96.6), LR 94.4% (84.4–100), LT 81.8% (61.9–100) (p = 0.43), 10-year RFA 80.1% (70.1–91.6), LR 85.9% (69.0–100), LT 71.6% (48.8–100) (p = 0.52), 15-year RFA 70.3% (56.0–88.2), LR 42.9% (15.7–100), LT 71.6% (48.8–100) (p = 0.93) (Fig. 2). Further, on multivariable analysis for ITT survival all modalities had an equivalent mortality hazard (ref: RFA, LR HR:1.13, 95%CI 0.33–3.82; p = 0.80 and LT HR 1.39, 95%CI 0.35–5.44; p = 0.60) (Table 2).

**Table 2:** Effect of treatment (RFA, LR, or LT) on intention-to-treat survival and disease-free survival.

Reference: RFA			
Outcome		HR (95% CI)	p-value
Intention-to-treat survival^a^			
	LR	1.13 (0.33–3.82)	0.80
	LT (listing)	1.39 (0.35–5.44)	0.60
Disease-free survival^a^			
	LR	0.52 (0.26–1.02)	0.06
	LT (transplant)	0.15 (0.03–0.67)	0.01

**Abbreviations:** CI: confidence interval, HR: hazard ratio, LR: liver resection, LT: liver transplant, RFA: radiofrequency ablation.

a Adjusted for a) patient characteristics: Model for End-Stage Liver Disease (MELD), age, b) tumor characteristics: tumor size, alpha-fetoprotein (AFP) tumor location and temporal trends: year of treatment.

### 3.3 Disease-free survival

**Fig. 3:**
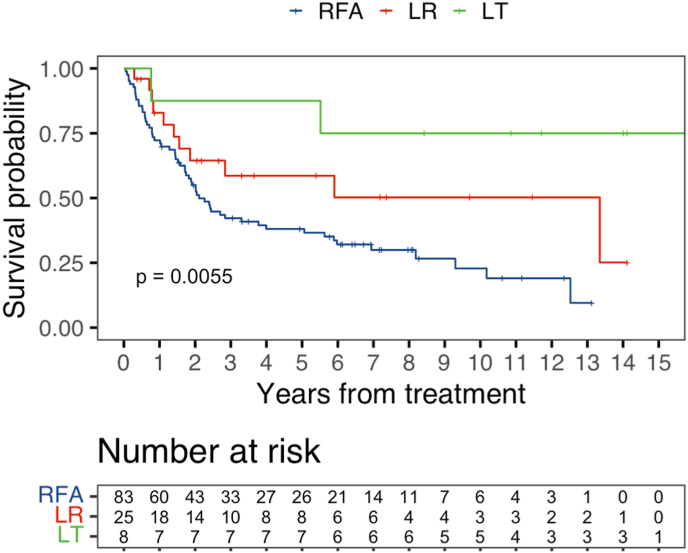
DFS overall cohort.

DFS was better overall in LT compared with RFA (p = 0.02), but not statistically significantly different than LR (p = 0.14) (Fig. 3). LR had DFS that was not statistically significantly different from the RFA group (p = 0.07). The median DFS was 2.1 years in the RFA group, 13.4 years in the LR group, and not reached in the LT group. The unadjusted DFS for 1-, 5-, and 10-year were: (%, 95% CI) 1-year RFA 72.3% (63.3–82.6), LR 82.9% (69.0–99.7), LT 87.5% (67.3–100) (p = 0.36), 5-year RFA 38.1% (28.7–50.4), LR 58.6% (40.9–84.1), LT 87.5% (67.3–100) (p = 0.03), and 10-year RFA 22.8% (13.6–38.4), LR 50.2% (31.4–80.4), and LT 75.0% (50.3–100) (p = 0.02). On multivariable analysis, relative to RFA, LR was associated with a non-statistically significantly different hazard of DFS, whereas the DFS hazard was better in the LT group (LR HR:0.52, 95% CI 0.26–1.02; p = 0.06; LT HR:0.15, 95% CI 0.03–0.67; p = 0.01). Though numerically lower, but not reaching statistical significance, LT was associated with a better DFS than LR (ref: LR, LT HR:0.30, 95% CI 0.06–1.43; p = 0.13) (Table 2**)**.

## 4 Discussion

The oncologic outcomes of the various treatment strategies for solitary HCC ≤ 3 cm are distinct. ITT survival is similar between the three modalities, however, adjusted DFS is similar in LR and LT, but better relative to RFA. These findings may offer impetus for future randomized prospective trials. In the interim, the differences in DFS can be factored into individualized treatment selection based on provider experience, as well as individual patient characteristics, wishes, and expectations.

RFA, LR, and LT are viable therapeutic options for very early and early-stage HCC [15,23]. Nonetheless, selecting the treatment modality of choice for these patients takes into account tumour burden, degree of cirrhosis, hepatic function, as well as the patients' functional status. Additionally, any clinical decision must balance best evidence-based medicine practices with patient values and preferences as well as clinical expertise. Staging systems, such as the BCLC, can help inform clinical decision-making, but cannot substitute for integration of all these factors, particularly given the geographic heterogeneity leading to notable variability in the clinical management of HCC [24]. Our findings share concordance with previous work identifying a therapeutic hierarchy, in order of survival benefit especially regarding DFS: LT, LR, ablation, intra-arterial therapies, systemic therapy, and best supportive care [25]. Nonetheless, a similar ITT survival was noted for each of the treatment modalities in our study. The difference may be in part to the outcome selected (ITT survival) as well as the specific solitary HCC ≤ 3 cm subgroup of patients.

The debate of LT versus LR currently only exists for the subgroup of patients with early-stage HCC – unifocal lesion < ∼5 cm and those with well-compensated cirrhosis without portal hypertension [5,8]. Both treatments have been proven to offer long-term survival in well-selected patients. Shah et al. from UHN and the University of Toronto assessed a cohort of 347 patients receiving LR (n = 174) and LT (n = 173) between 1995 and 2005 [9]. The overall survival (from LR or LT listing), analyzed with an intention-to-treat principle, was equivalent between the two groups (1-,3-,5- year LR 89%, 75%, 56% vs. LT 90%, 70%, 64%; p = 0.84) [9]. The group also identified that a prolonged (>4 month) waitlist time portended a higher risk of death (OR 2.5, 95% CI 1.3–5; p = 0.007), and concluded that unless waitlist time for an LT is short (<4 months), either treatment option (LR or LT) can be considered in patients with early HCC (defined as within Milan criteria) and adequate hepatic reserve [9]. With regards to recurrence, LR has been associated with a lower recurrence-free survival than LT, with 5-year recurrence exceeding 50% following LR compared to 10–20% after LT [5,26,27]. However, due to the scarcity of organs available for transplantation as well as associated wait times and high costs associated with LT, both LR and RFA have become the preferred options for first-line management in eligible patients. Additionally, in certain jurisdictions, including the United Network for Organ Sharing (UNOS), patients with T1 tumours (1 lesion < 2 cm) are not eligible for priority listing for LT, mostly secondary to a low risk of dropout while on the waitlist and chance for HCC misdiagnosis [28]. This has led to two clinical practices; one is to immediately treat these tumours with locoregional therapies (typically RFA) [29]. An alternative strategy is to wait, without performing any locoregional treatment, until the tumour progresses to a T2 (one lesion 2–5 cm or 2-3 lesions ≤ 3 cm) to achieve eligibility for listing, with MELD exception points. Predictors of rapid progression included Hispanic ethnicity and alcohol-related cirrhosis [28]. Patients with a high risk for waitlist dropout had AFP ≥500 ng/mL and accelerated tumour progression, and are those who may rather benefit from early locoregional therapy [28].

Locoregional tumour therapies, such as radiofrequency ablation, afford high tumour response and acceptable survival, but with high recurrence rates. Rossi et al. evaluated 706 patients with Child-Pugh A and B8 cirrhosis with 859 HCC lesions ≤3.5 cm [30]. The cumulative incidence of the first recurrence at 3- and 5-years was 70.8% and 81.7%, respectively. The 3- and 5- year OS (after repeated RFAs) was 67.0% and 40.1%, respectively. Despite the high recurrence, RFA is safe and effective in HCC disease control in cirrhotic patients and offers the ability for treatment repetition in the case of intrahepatic recurrence [30]. In a systematic review and meta-analysis comparing RFA and LR, Xu et al. showed that RFA is associated with lower complications and shorter hospitalization [11]. Compared to LR, RFA had a higher recurrence rate but with similar OS. Based on a trial sequential analysis, over 10,000 patients would be needed to prove a significant difference in 3-year survival between these two treatment modalities [11]. Resection outcomes are also sensitive to liver function. Chong et al. from Hong Kong evaluated the survival of patients with HCC who received either LR or microwave ablation and the utility of the Albumin-Bilirubin (ALBI) [31] score in selecting patients for treatments. In their retrospective analysis of 442 patients (LR n = 379, MWA n = 63), 63 pairs of patients were propensity matched on demographic and clinicopathologic variables [32]. Patients who underwent LR had a better OS and DFS if the ALBI grade was 1 (3-year survival LR 82.6% vs. MWA 72.3%; p = 0.19) whereas MWA had a better OS (3-year survival LR 54.9% vs. 71.5%; p = 0.03) with similar DFS in patients with ALBI scores of 2 or 3 [32]. Consequently, the group proposed that ALBI grade, as an assessment of liver function, be incorporated in the decision-making process for these treatment modalities [32]. Moreover, ablation outcomes are sensitive to tumour size, as demonstrated by Kutlu et al. in an analysis of the SEER database between 2004 and 2013, where no difference was noted in survival for LR vs. RFA if HCC ≤ 3 cm. In contrast, LR was superior once tumours exceeded three cm [7]. In a cost-effectiveness analysis comparing LR vs. RFA for early-stage HCC by Cucchetti et al. for very early HCC in Child-Pugh A patients, RFA achieved similar quality-adjusted life-years (QALY) at lower costs than LR [33]. For solitary lesions between 3 and 5 cm, LR offered better life expectancy and better cost-effectiveness than RFA [33].

Apart from patient demographic and clinicopathologic variables, other factors can influence the choice of therapy. These include local expertise and patient preference (with considerations for procedural invasiveness, anticipated hospital length of stay, and morbidity and mortality risks). The role of laparoscopic LR within this context will need to be explored and is an approach that has been shown to result in lower blood loss, lower transfusion rates, shorter length of stay, fewer postoperative complications, without differences in the excised surgical margin, positive margin resection rates, or tumour recurrence [34]. Tumour location, such as proximity to vascular structures, represents a variable that also needs to be considered during treatment selection. Lee et al. evaluated LR and RFA as first-line treatment in patients with perivascular (defined as tumour abutting the first- or second-degree branches of a portal or hepatic vein) HCC ≤ 3 cm, within BCLC stage 0 or A and found that after propensity matching, extrahepatic recurrence and OS was better in the LR group compared to the RFA group for patients with periportal HCC, however, extrahepatic recurrence and OS were similar in patients with perivenous HCC [35]. Besides the size, the location of the tumour in the liver is a critical factor that dictates the extent of resection that will be required. In a patient with underlying liver disease, a deep lesion that may require a more extensive hepatic resection may be more likely to be recommended to undergo an LT or RFA. To account for this potential confounding, we included tumour location in the multivariable adjustments. Anatomic considerations are also important with regards to locoregional therapies, as insufficient ablation near vascular structures has also been attributed to a phenomenon known as the “heat-sink” effect, whereby during RFA heat loss occurs into hepatic vessels adjacent to the tumour and influence the efficacy of ablation [36,37]. It has thus been suggested that both LR and RFA can be considered as first-line for perivenous HCC, but that LR be preferred in periportal HCC [35].

This study is limited by its single-institutional and retrospective nature, with the potential for misclassification and selection bias. We have attempted to overcome some of the selection bias by including only patients who were eligible for all treatments to allow for a clinically relevant comparison between the groups. Nonetheless, there is potential for residual and unmeasured confounding. Given the small sample size, inferences and generalizability are limited, and the potential for type II error should thus be recognized. Moreover, some patients may have been recommended to undergo one treatment modality over another based on factors that have not been objectively accounted for such as difficulty with resection or image-guided ablation. Finally, recognizing the challenges inherent to a randomized controlled trial in this setting, a prospective evaluation of patients deemed eligible for all three treatment modalities and receiving counseling therein may offer insight into oncologic outcomes with less potential for selection bias than a retrospective evaluation.

## 5 Conclusions

In conclusion, for patients with solitary HCC ≤ 3 cm, the intention-to-treat survival for those receiving RFA, LR, or LT are equivalent, with adjusted DFS of either LR or LT being better relative to RFA. Taken into combination, the demonstrated equipoise between treatment modalities supports a potential prospective trial for patients truly eligible for all treatments.

## Ethical approval

This study was approved by our institutional Research Ethics Board and Alan Barolet, MD, PhD FRCPC Co-Chair at University Health Network and a waiver of informed consent was obtained. The relevant Judgement’s reference number is **16-5285**

## Sources of funding

None

## Author contribution

**TI:** Conception of project, literature review, interpretation of results and write-up of the manuscript, **LR:** Literature review, data analysis, interpretation of results, write up of the manuscript, **PA:** Conception of project, write up of the manuscript, **MC:** Conception of project, data analysis, interpretation of results, write up of the manuscript, **CS:** Conception of project, data analysis, interpretation of results, write up of the manuscript, **MP:** Conception of project, data analysis, interpretation of results, write up of the manuscript, **WJC:** Conception of project, data analysis, interpretation of results, **AD:** Conception of project, data analysis, interpretation of results, **HM:** Conception of project, data analysis, interpretation of results, **IM:** Conception of project, interpretation of results, **MS:** Conception of project, interpretation of results, **RB:** Conception of project, interpretation of results, **JK:** Conception of project, interpretation of results, **MB:** Conception of project, interpretation of results, **NS:** Conception of project, interpretation of results, **AG:** Conception of project, interpretation of results, **MC:** Conception of project, interpretation of results, **BS:** Conception of project, interpretation of results, **TR:** Conception of project, interpretation of results, **LL:** Conception of project, data analysis, interpretation of results, **ZG:** Conception of project, data analysis, interpretation of results, **GS:** Conception of project, data analysis, interpretation of results, literature review, write up of the manuscript

## Consent

This study was approved by our institutional Research Ethics Board (REB #16-5285), and a waiver of informed consent was obtained.

## Trial registry number

Name of the registry: ClinicalTrials.gov PRS
Unique Identifying number or registration ID: NCT05193253
Hyperlink to your specific registration (must be publicly accessible and will be checked): https://clinicaltrials.gov/ct2/show/NCT05193253?term=NCT05193253&draw=1&rank=1

## Guarantor

Gonzalo Sapisochin

## Provenance and peer review

Not commissioned, externally peer-reviewed.

## Declaration of competing interest

Gonzalo Sapisochin discloses consultancy for Aztra-Zeneca, Roche, Novartis, and Integra. Gonzalo Sapisochin has received financial compensation for talks for Roche, Aztra-Zeneca, Chiesi, and Integra. Gonzalo Sapisochin has received a grant from 10.13039/100004337Roche. None of the other authors have any conflicts of interest to declare.
