# Supplementary material for: Long-term outcomes of ablation, liver resection, and liver transplant as first-line treatment for solitary HCC of 3 cm or less using an intention-to-treat analysis: A retrospective cohort study
Source: Ann Med Surg (Lond). 2022 Apr 20;77:103645. doi: 10.1016/j.amsu.2022.103645 (PMC9142643; doi:10.1016/j.amsu.2022.103645)
Supplement: Multimedia component 2 [file mmc2.docx]

## **Table S1: Additional information for patients listed for LT**

| **Listed patient number** | **Reason for LT listing** | **Drop-out reason** | **Listing date** | **Transplant date** | **Recurrence** | **Recurrence details** | **Death** |
| --- | --- | --- | --- | --- | --- | --- | --- |
| **1** | HBV+HCC with well-compensated liver function, all options presented to the patient | - | Sep-2005 | Oct-2005 | No | - | Yes |
| **2** | HCV+HCC, well-compensated liver function (noted to have portal hypertension, splenomegaly, varices, some cirrhosis), all options presented, expected mortality with resection >20%, LT was recommended given degree of portal hypertension | Died | Oct-2006 | Dropout | No | - | Yes |
| **3** | RFA vs. LT, resection not offered given advanced liver disease (noted to have mild cirrhosis, portal hypertension, no splenomegaly) | - | Jul-2006 | Apr-2007 | Yes | Mediastinal metastasis | Yes |
| **4** | Hepatitis cirrhosis+HCC, no coagulopathy but due to cirrhosis the patient was not considered a suitable candidate for LR, was treated with ablation as an option to bridge to LT | - | Jun-2002 | May-2004 | No | - | No |
| **5** | No decompensation, moderate cirrhosis, near-complete left-sided atrophy and thrombocytopenia, LR undesirable option, ablation offered as definitive vs. bridging option, patient chose transplant | - | May-2007 | Jul-2007 | No | - | No |
| **6** | HCV+HCC, no decompensation, grade 2 varices on nadolol, options discussed, LR considered high-risk due to portal hypertension (based on varices) and thrombocytopenia, definitive ablation vs. ablation as bridge to LT seen as best options | Control of disease with ablation | June-2007 | Dropout | No | - | No |
| **7** | HBV+HCC into caudate lobe, indenting inferior vena cava, well-compensated liver disease, multidisciplinary discussion, given location not considered ablatable or surgically resectable, LT recommended | - | Jan-2004 | Apr-2004 | No | - | No |
| **8** | All three options discussed, all considered appropriate, LT chosen with expectation to yield optimal long-term outcomes | - | Mar-2007 | Sep-2007 | No | - | No |
| **9** | HCC in cirrhotic liver, all options discussed, seemed resectable initially, however on reassessment would require right hepatectomy and given presence of varices LT considered to be better option | Disease control | Nov-2005 | Dropout | No | - | No |
| **10** | HBV+HCC, no decompensation, some cirrhosis, tumor intimate to inferior vena cava, may have been resectable initially though due to relationship to inferior vena cava LT considered to be better option | - | Feb-2009 | Apr-2009 | No | - | No |
| **11** | HBV+HCV and HCC, no decompensated liver function, no ascites or varices, Childs-Pugh A, no portal hypertension, platelet count >200,000, all three options presented | - | Jul-2015 | Dec-2015 | Yes | Solitary portocaval adenopathy and intra-hepatic multifocal recurrence | Yes |
| **Abbreviations:** HBV: hepatitis B virus, HCV: hepatitis C virus, HCC: hepatocellular carcinoma, LT: liver transplant, LR: liver resection, RFA: radiofrequency ablation, | | | | | | | |
